# Multisequence combined magnetic resonance imaging radiomics model to noninvasively predict nuclear grade of clear cell renal cell carcinoma: interpretable model development

**DOI:** 10.1590/1806-9282.20241012

**Published:** 2025-03-17

**Authors:** Esat Kaba, Hande Melike Bülbül, Mehmet Kıvrak, Nur Hürsoy

**Affiliations:** 1 Recep Tayyip Erdogan University, Department of Radiology – Rize, Turkey.; 2 Recep Tayyip Erdogan University, Department of Biostatistics and Medical Informatics – Rize, Turkey.

**Keywords:** MRI, Radiomics, Nuclear grade, Renal cell carcinoma, Machine learning

## Abstract

**OBJECTIVE:**

The nuclear grade of clear cell renal cell carcinoma directly relates to prognosis and is usually determined through invasive methods like biopsy or surgery. This study aimed to predict the nuclear grade of clear cell renal cell carcinoma using a noninvasive method: multisequence magnetic resonance imaging-based radiomics analysis.

**METHODS:**

A total of 42 clear cell renal cell carcinomas (29 low grade, 13 high grade) were included in the study. T2, fat-suppressed T2, noncontrast T1, corticomedullary phase, nephrographic phase, excretory phase, and apparent diffusion coefficient sequences of patients were used for radiomics analysis. Inter-observer agreement was assessed for these sequences, and following reproducibility analysis and feature selection, three new groups were formed: noncontrast enhancement, contrast enhancement, and combined groups, with different combinations of features extracted from these sequences. As a result, seven different sequences and three different groups constituted 10 classification groups. An extreme gradient boosting model was used for classification, employing 10-fold cross-validation.

**RESULTS:**

Radiomics features from corticomedullary phase and nephrographic phase sequences showed excellent inter-observer agreement, with Pearson correlation coefficient values of 0.88 for corticomedullary phase and 0.90 for nephrographic phase. The study included 42 clear cell renal cell carcinomas with a mean age of 60.8 years. Individually, the corticomedullary phase sequence achieved the highest area under the curve and accuracy values (0.88 and 0.85), followed by the apparent diffusion coefficient sequence (0.87 and 0.79). In the combined sequence group, the contrast enhancement group showed the highest area under the curve and accuracy (0.93 and 0.87), ranking highest across the entire study.

**CONCLUSION:**

Multisequence magnetic resonance imaging radiomics has great potential to predict the nuclear grade of clear cell renal cell carcinoma and guide the treatment plan noninvasively.

## INTRODUCTION

Renal cell carcinoma (RCC) accounts for 2.2% of all newly diagnosed cancers worldwide^1^. The World Health Organization (WHO) classification of renal tumors is based on a combination of morphological, molecular, and genetic features. The most common is the clear cell type (70–90%), followed by papillary (10–15%) and chromophobe RCCs (3–5%). Clear cell RCC (ccRCC) constitutes the most common subtype with approximately 80%, and its prognosis is worse than other subtypes^2,3^.

The most relevant prognostic factors for ccRCC are tumor stage and WHO/International Society of Urologic Pathologists (ISUP) nuclear grade^4^. CcRCCs consist of four grades based on nuclear morphology; grades 1 and 2 are considered low grade, while grades 3 and 4 are regarded as high grade, which are directly associated with poor prognosis^5,6^. Computed tomography (CT) and magnetic resonance imaging (MRI) are used in noninvasive preoperative evaluation, and biopsies are generally used to determine the preoperative nuclear grade^7^. However, even with advanced technological techniques such as MRI wash-in index and diffusion kurtosis tensor MRI, imaging findings often overlap between low and high groups^8–11^. Biopsy is an invasive method with limitations such as sampling site inaccuracy and bleeding complications. There is also a very small risk of tumor seeding^12^. Since it can potentially change the treatment modality, it is important to determine the preoperative nuclear grade. For this purpose, some noninvasive techniques are currently being investigated. Radiomics studies are at the forefront of these^12–14^.

In the literature, authors have generally focused on CT-based radiomics studies to predict the nuclear grade of ccRCC. Although there are some MRI-based studies, in this study, we aimed to predict the nuclear grade of ccRCC with a wide range of sequences and their combinations, different from the studies in the literature. In addition to the literature, we compared the performance of radiomics analysis of different noncontrast MRI sequences and contrast-enhanced MRI sequences in predicting nuclear grade.

## METHODS

### Data set

Patients who underwent surgery for renal mass from 2017 to 2022 in our hospital and whose pathology result was ccRCC were included in the study. Our inclusion criteria were (1). patients with pathologically proven ccRCC, (2) patients with imaging within 1 month before surgery, (3) patients scanned on a 3 T MRI scanner, and (4) patients with dynamic contrast MRI. Our exclusion criteria were patients without MRI, patients obtained from a 1.5 T MRI scanner, and other tumors other than ccRCC. After applying the exclusion criteria, 42 ccRCCs from 39 patients were included in the study. The nuclear grades of all patients were histopathologically proven after partial or total nephrectomy. All pathological examinations were conducted by a pathologist experienced in genitourinary system pathology. Pathology results were collected from the hospital record system. Nuclear grades 1 and 2 were defined as low grades and 3 and 4 as high^15^.

This study was performed in line with the principles of the Declaration of Helsinki. Approval was granted by the Ethics Committee of our hospital (2024/19).

### Radiomics analysis

**Figure 1:**
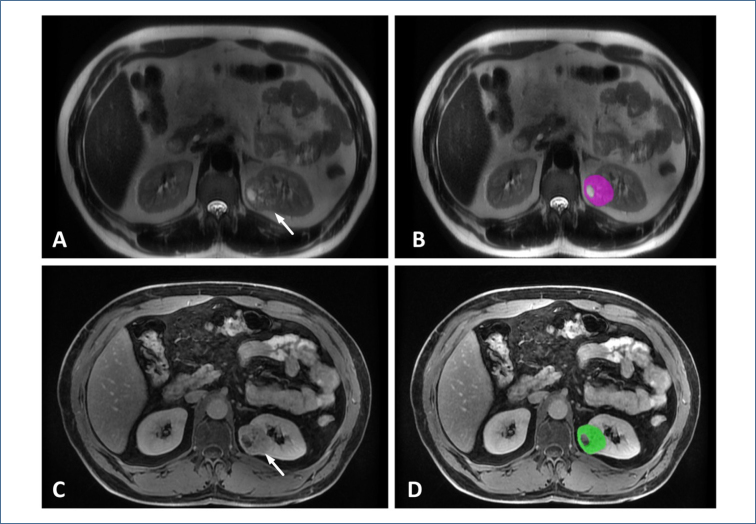
Clear cell renal cell carcinoma in the left kidney (arrows). (A) T2 sequence, (B) region of interest drawing excluding gross necrotic area, (C) excretory phase sequence, and (D) region of interest drawing excluding gross necrotic area.

T2, fat-suppressed T2 (T2-FS), noncontrast T1, corticomedullary phase (CMP), nephrographic phase (NP), excretory phase (EP), and apparent diffusion coefficient (ADC) sequences of patients were used for radiomics analysis. The tumor was segmented using the freely available LIFEx software (version 7.2.10) (www.lifexsoft.org). Two radiologists with 4 and 8 years of abdominal imaging experience independently analyzed the images. The observers were blinded to the patient's pathological data. A 2D region of interest (ROI) was drawn in the widest part of the tumor, avoiding necrotic and hemorrhagic areas (Figure 1). To ensure uniformity for texture analysis, gray-level discretization was performed by specifying a gray-level range between 1 and 128 bits/pixel. Intensity rescaling values were assigned between mean±3 standard deviations. Dimension processing was performed in the 2D axial plane. Morphological features, first-order features (Intensity_based and Intensity_histogram), and second-order features (gray-level co-occurrence matrix, neighborhood gray-level difference matrix, gray-level run length matrix, gray-level zone length matrix) were extracted.

### Reproducibility analysis and feature selection

Intraclass correlation coefficient (ICC), Pearson correlation coefficient (PCC), and R^2^ were calculated to analyze intra-observer variability. ICC>0.75 was considered good reproducibility^16^. Feature reduction was performed with the least absolute reduction and selection operator (LASSO) regression model on the obtained radiomics features.

### Machine learning

In the study, ccRCC grading prediction was first performed using seven different sequences mentioned above. Then, the contrast-enhanced sequences (CMP, NP, EP) were combined and the "contrast enhancement (CE)" group was created. The remaining four sequences without contrast material (T2, T2 FS, T1, ADC) were combined to form the "noncontrast enhancement (NCE)" group. In addition, a "combined" group was created in which all sequences were used. As a result, grading prediction for ccRCC was performed with 10 different data sets, including 7 different sequences and 3 groups created with them. Since fewer tumors existed in the high-grade group, the synthetic minority over-sampling technique (SMOTE) was applied to eliminate the imbalance between the groups, and the number of both groups was equalized. Then, the extreme gradient boosting (XGBoost) algorithm was used for classification. The 10-fold cross-validation method was used for classification. Performance was evaluated by area under the curve (AUC), accuracy, sensitivity, specificity, F1 score, positive predictive value (PPV), and negative predictive value (NPV). All procedures were performed in the Jupyter Notebook environment with Python 3.9.

## RESULTS

Radiomics features obtained from CMP and NP sequences were analyzed to assess inter-observer agreement. For the CMP sequence, PCC was 0.88, ICC was 0.78, and R^2^ was 0.74, while for the NP sequence, PCC was 0.90, ICC was 0.80, and R^2^ was 0.80. These results show an excellent correlation between both observers.

The study included 42 ccRCCs of 39 patients. Also, 10 of the patients were female and 29 were male with a mean age of 60.8 (36–80) years. When each sequence was evaluated individually, CMP showed the highest AUC and accuracy values with 0.88 and 0.85, respectively, followed by the ADC sequence with 0.87 and 0.79. In the combined sequence group, the highest AUC and accuracy values were in the CE group, with 0.93 and 0.87, respectively. The CE group also achieved the highest AUC and accuracy values in the entire study. All performance metrics are given in Table 1.

**Table 1:** All performance metrics.

	AUC	Accuracy	Sensitivity	Specificity	F1 score	PPV	NPV
T2	0.78	0.78	0.86	0.69	0.79	0.74	0.83
T2 FS	0.83	0.76	0.72	0.79	0.75	0.78	0.74
T1	0.73	0.63	0.78	0.48	0.68	0.60	0.69
CMP	0.88	0.85	0.92	0.77	0.86	0.80	0.91
NP	0.82	0.76	0.85	0.67	0.78	0.72	0.82
EP	0.82	0.72	0.76	0.68	0.73	0.70	0.74
ADC	0.87	0.79	0.82	0.75	0.79	0.77	0.81
NCE	0.86	0.80	0.86	0.74	0.81	0.77	0.85
CE	0.93	0.87	0.90	0.84	0.87	0.85	0.89
All	0.91	0.82	0.84	0.80	0.82	0.81	0.83

AUC: area under the curve; PPV: positive predictive value; NPV: negative predictive value; CMP: corticomedullary phase; NP: nephrographic phase; EP: excretory phase; T2 FS: fat-suppressed T2; ADC: apparent diffusion coefficient; NCE: noncontrast enhancement; CE: contrast enhancement.

**Figure 2:**
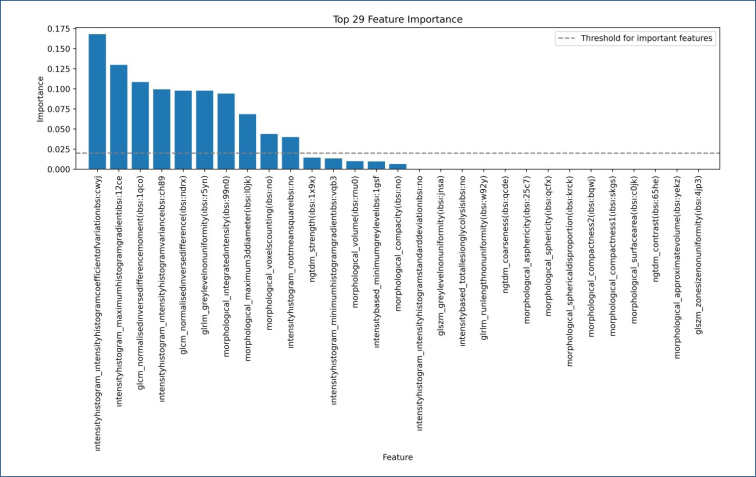
Feature importance plot shows the contribution of features to the machine learning model's results. While 15 features affect the model's performance, 14 have no impact on the model results obtained in our study.

The features selected with LASSO and their importance according to the XGBoost model are shown in Figure 2. Here, the features that affect the predictive power of the XGBoost model and their power of influence are shown. According to this, it is seen that 14 of the 29 features do not affect the model's performance as negative or positive.

## DISCUSSION

A strong correlation exists between prognosis and WHO/ISUP nuclear grade in patients with ccRCC. Preoperative grading can significantly affect treatment modality as higher grades correlate with worse prognosis^17^. In patients with comorbidities and the elderly, follow-up may be preferred over major surgical interventions for low-grade tumors^18^. Although percutaneous biopsy is used for grading, it has limitations and complications, such as difficulty representing the whole tumor and risks of bleeding, infection, pseudoaneurysm, and AV fistula development^19,20^. Consequently, radiomics studies have emerged as a potential alternative to biopsy.

Most radiomics studies are CT-based. Lin et al. reported the highest AUC of 0.87 in a grade prediction study with three-phase CT^19^. Xv et al. achieved the highest success using the SVM algorithm with combined clinical–radiological and radiomics models, obtaining train, validation, and test AUCs of 0.887, 0.859, and 0.828, respectively^6^. Yin et al. showed the highest performance with the Random Forest model using noncontrast phase CMP, NP, excretory phases, and all-phase, with a test set AUC value of 0.89^21^.

Compared to CT-based studies, MRI-based grade prediction studies for ccRCC are relatively rare. This may be due to the higher accessibility and faster acquisition of CT, leading to its preferred use for RCC diagnosis in some centers. Moreover, the extensive range of sequences in MRI can make it more time-consuming for radiomics analyses. Goyal et al. used 1.5 T MRI in a study of 34 ccRCCs, achieving an AUC of 0.889 with the CMP sequence^12^. Stanzione et al. used 3 T MRI with 32 patients, employing T2 and arterial phase sequences with SMOTE and J48 decision tree classification^22^. They reported over 90% accuracy for the ensemble model and 84.4% with the random forest model alone. Cui et al. included T1w, T2w, CMP, and NP sequences, reporting the highest accuracies as 64, 69, 69, and 68%, respectively, and 74% accuracy when combining all sequences^23^.

Compared with the studies in the literature, our study includes the most comprehensive set of sequences to our knowledge. The 93% AUC value we achieved is competitive with literature reports. Our study uniquely develops an interpretable machine-learning model, simulating a radiologist's daily RCC evaluation. The high success rate in the CMP sequence and CE group aligns with the vascular nature of ccRCC. Additionally, the ADC sequence showed a sensitivity of 0.82, likely due to differences in cellularity between low- and high-grade tumors.

There were some limitations to our study. First, the number of patients was small due to the strict exclusion and inclusion criteria. We tried to overcome this with SMOTE and eliminate the imbalance between the classes. Future studies with similar sequence diversity and larger numbers of patients may overcome this limitation. Second, we performed radiomics analysis with 2D MRI images. In the future, 3D analysis representing the whole tumor can be performed with all sequences.

In conclusion, multisequence MRI radiomics has great potential to predict the nuclear grade of ccRCC and guide the treatment plan noninvasively. This study used an interpretable machine-learning model to reveal the background of successful predictions. The results show that radiomics analysis, especially of contrast-enhanced sequences, holds promise for future use in predicting the nuclear grade of ccRCC. Thus, radiomics analysis could reduce the number of biopsies and further expand the role of preoperative imaging in the treatment plan.
